# CD18 deficiency improves liver injury in the MCD model of steatohepatitis

**DOI:** 10.1371/journal.pone.0183912

**Published:** 2017-09-05

**Authors:** Andrew A. Pierce, Caroline C. Duwaerts, Kevin Siao, Aras N. Mattis, Amanda Goodsell, Jody L. Baron, Jacquelyn J. Maher

**Affiliations:** 1 Department of Medicine, University of California, San Francisco, San Francisco, California, United States of America; 2 Liver Center, University of California, San Francisco, San Francisco, California, United States of America; 3 Department of Pathology, University of California, San Francisco, San Francisco, California, United States of America; Institute of Medical Research A Lanari-IDIM, University of Buenos Aires-National Council of Scientific and Technological Research (CONICET), ARGENTINA

## Abstract

Neutrophils and macrophages are important constituents of the hepatic inflammatory infiltrate in non-alcoholic steatohepatitis. These innate immune cells express CD18, an adhesion molecule that facilitates leukocyte activation. In the context of fatty liver, activation of infiltrated leukocytes is believed to enhance hepatocellular injury. The objective of this study was to determine the degree to which activated innate immune cells promote steatohepatitis by comparing hepatic outcomes in wild-type and CD18-mutant mice fed a methionine-choline-deficient (MCD) diet. After 3 weeks of MCD feeding, hepatocyte injury, based on serum ALT elevation, was 40% lower in CD18-mutant than wild-type mice. Leukocyte infiltration into the liver was not impaired in CD18-mutant mice, but leukocyte activation was markedly reduced, as shown by the lack of evidence of oxidant production. Despite having reduced hepatocellular injury, CD18-mutant mice developed significantly more hepatic steatosis than wild-type mice after MCD feeding. This coincided with greater hepatic induction of pro-inflammatory and lipogenic genes as well as a modest reduction in hepatic expression of adipose triglyceride lipase. Overall, the data indicate that CD18 deficiency curbs MCD-mediated liver injury by limiting the activation of innate immune cells in the liver without compromising intrahepatic cytokine activation. Reduced liver injury occurs at the expense of increased hepatic steatosis, which suggests that in addition to damaging hepatocytes, infiltrating leukocytes may influence lipid homeostasis in the liver.

## Introduction

Non-alcoholic steatohepatitis (NASH) is a disease characterized by accumulation of fat within hepatocytes accompanied by liver injury and inflammation. NASH occurs in approximately 6% of the population [1] and accounts for as much as two-thirds of the unexplained liver disease in the United States [2, 3] Inflammatory pathways play a central role in the development of NASH; Toll-like receptors (TLRs) on liver cells sense a variety of danger signals from within and outside the liver and cooperate with inflammasomes to induce liver cell death, inflammation and fibrosis [4–6] In many experimental models of NASH, the pivotal cells responsible for initiating liver disease are the Kupffer cells. These liver-resident macrophages regulate many of the features of NASH by secreting cytokines and chemokines that enhance hepatic steatosis, recruit circulating leukocytes to the liver, and stimulate collagen production by hepatic stellate cells [4, 5]. Once this complex series of events is set in motion, however, the exact contribution of recruited inflammatory cells to NASH is difficult to dissect. The objective of this study was to identify the specific impact of recruited leukocytes on the development of NASH in an animal model.

CD18, also known as β_2_-integrin, is a leukocyte adhesion molecule that plays a role in the transendothelial migration of leukocytes into sites of tissue injury. CD18 is expressed primarily on cells of the granulocyte lineage (neutrophils, monocytes and macrophages), where it forms heterodimers with β-integrin subunits (CD11a, b, c, and d) and facilitates binding of leukocytes to intercellular adhesion molecules on the surface of target cells [7, 8]. A mutation in the human CD18 gene (ITGB2) leads to a condition called leukocyte adhesion deficiency, which is characterized by neutrophilia and impaired neutrophil recruitment to sites of infection [7]. Mice with a mutation in the CD18 gene also have neutrophilia and impaired leukocyte emigration in experimental peritonitis and other inflammatory diseases [9–12].

Based on the above understanding of CD18 biology and NASH-related inflammation, we hypothesized that CD18 deficiency would protect mice from hepatic inflammation and liver injury in an experimental model of steatohepatitis. NASH can be induced in mice by feeding them an energy-rich, methionine-choline-deficient (MCD) diet for 3 weeks. MCD-mediated steatohepatitis develops rapidly in mice and bears close histologic resemblance to human NASH; [13–15] importantly, hepatic inflammation is pronounced in MCD-fed mice [16–18], and thus the model offers a platform for assessing the effect of infiltrating leukocytes on overall liver outcome.

Our results demonstrated that CD18 deficiency did not inhibit the hepatic cytokine activation characteristic of early NASH in MCD-fed mice. Nor did it impair leukocyte migration to the liver in response to MCD feeding. CD18 deficiency did, however, significantly reduce liver injury in MCD-fed mice, because of defective activation of recruited leukocytes. Unexpectedly, CD18-mutant mice developed more hepatic steatosis than wild-type mice in response to MCD feeding despite being spared from steatohepatitis. The reason for this is unclear, but persistent Kupffer cell activation, impaired triglyceride lipolysis and reduced activity of recruited leukocytes may all play contributory roles.

## Materials and methods

### Animals and diets

Adult male mice (21–25 g) expressing a mutant CD18 (*Itgb2*^*tm1Bay*^) on a C57BL/6 background were bred from a colony provided by Dr. Arthur Beaudet [9]. Wild-type C57BL/6 controls (WT) of the same gender and weight were purchased from the Jackson Laboratory (Bar Harbor, ME). Mice were housed in groups of 3–5 with free access to food and water and a 12-h light/dark cycle. Weight-matched cohorts were fed either a chow diet (#5053; LabDiet, Richmond, IN) or an MCD formula (#518828; Dyets, Bethlehem, PA) for 3–8 weeks. At the end of each experiment, mice were fasted for 4 h and killed at approximately 12N by exsanguination under deep isoflurane anesthesia. All procedures were approved by the UCSF Institutional Animal Care and Use Committee.

### Serum chemistries

Alanine aminotransferase (ALT) was assayed on an ADVIA 1800 autoanalyzer (Siemens Healthcare Diagnostics, Deerfield, IL) in the clinical chemistry laboratory at San Francisco General Hospital.

### Histology and immunohistochemistry

Formalin-fixed liver sections were stained with H&E. Slides were reviewed blindly by a certified pathologist and scored for steatosis, ballooning and inflammation as described by Kleiner et al [19]. Granulocytes were identified in frozen sections of liver tissue using an antibody against Gr-1 (Ly6C/G, BD Biosciences, San Jose, CA) as previously described [20] and counterstained with hematoxylin. Cleaved caspase-3 (#9664, Cell Signaling Technology, Danvers, MA) and chlorotyrosine-protein adducts (#HP5002, Hycult Biotech, Plymouth Meeting, PA, USA) were identified in formalin-fixed sections by immunohistochemistry following sodium citrate unmasking. Infiltrating leukocytes and caspase-3-positive cells were quantified by counting stained cells in 10 consecutive microscopic fields to generate a mean value per liver. Chlorotyrosine adduct abundance was quantified as the percentage of stained tissue in a minimum of 500 μm^2^ liver tissue. Hepatic collagen was highlighted by Sirius Red staining and quantitated similarly by morphometry (Image J; http://rsb.info.nih.gov/ij/).

### Hepatocyte isolation and culture

Primary mouse hepatocytes were isolated by retrograde perfusion of the liver with Liver Perfusion Medium (Life Technologies, Grand Island, NY), followed by Liver Digest Medium (Life Technologies) at 37°C for 4 minutes. Livers were then excised and minced in DME + 5% FBS to create crude cell suspensions, which were filtered through sterile gauze. Cells were pelleted from the suspension by two rounds of low-speed centrifugation (150g, 2 minutes each). Hepatocytes were purified from the washed pellets by resuspension in culture medium and centrifugation through 50% Percoll (GE Healthcare Life Sciences, Piscataway, NJ). Viability was > 90%. Purified hepatocytes were plated in DME/F12 containing 5% fetal bovine serum. Two hours after plating, the cells were washed and replenished with Williams E medium either with methionine and choline (MCS) or without methionine and choline (MCD) (Caisson Labs, Logan, UT). Cells were harvested at 48 h for measurement of cellular triglyceride.

### Isolation of liver immune cells

Mouse livers were perfused as described for hepatocyte isolation. When hepatocytes were separated from crude cell suspensions by low-speed centrifugation, the supernatants containing non-parenchymal cells were pelleted by centrifugation at 650*g* for 10 minutes. The 650*g* pellets were resuspended and separated on a 25%:50% Percoll gradient to isolate hepatic leukocytes.

## Flow cytometry

Cells were pre-incubated with FC/block (10mg/ml, 2.4G2, BD Biosciences) and stained according to standard protocols with combinations of the following anti-mouse antibodies: CD18-FITC (clone C71/16, #553292, BD Biosciences), CD11b-PerCP-Cy™5.5 (clone M1/70, #550993, BD Biosciences), F4/80-PE-Cy7 (clone BM-8, #25–4801, eBioscience, San Diego, CA), Ly-6C-AF700 (clone AL-21, #561237, BD Biosciences) and Ly-6G-PerCP-Cy™5.5 (clone 1A8, #560602, BD Biosciences). Cells were analyzed using an LSRII flow cytometer (BD Biosciences) and FlowJo software (Tree Star, Ashland, OR).

### Quantitation of hepatic triglyceride

Lipids were extracted from fresh liver tissue using the Folch method [21]. Samples were processed and analyzed for total triglyceride as previously described.[22] For measurement of triglyceride in cultured hepatocytes, cells were scraped into a neutral salt solution pelleted by centrifugation, and homogenized in 2:1 chloroform:methanol using the same method as that for whole tissue. Triglyceride was measured using a commercial kit (TR0100, Sigma Chemical Company, St. Louis, MO), and results were normalized either to liver weight (for whole tissue) or cellular protein (for cultured hepatocytes).

### Evaluation of gene expression by quantitative PCR

RNA was extracted from liver using TRIzol reagent (Life Technologies, Carlsbad, CA) and purified using the RNeasy kit (Qiagen, Valencia, CA). RNA integrity was verified by formaldehyde gel electrophoresis. cDNA was synthesized using iScript (BioRad, Hercules, CA); quantitative PCR (qPCR) was performed with TaqMan® assay kits (Life Technologies, Carlsbad, CA) using β-glucuronidase as the internal control gene.

### Analysis of hepatic triglyceride secretion

Mice fed MCD diets for 14 days were injected intraperitoneally with Tyloxapol **(**Sigma) as previously described [23]. Blood samples were obtained via submandibular venipuncture prior to injection and at 4 h and 8 h post-injection; serum triglyceride concentrations were measured as previously described [22].

### Myeloperoxidase assay

Myeloperoxidase activity was quantitated by using a tetramethylbenzidine (TMB)-based assay as previously described [24, 25]. Tissue samples were homogenized in phosphate buffer (20 mM, pH 7.4) and centrifuged (13,000*g*, 10 min, 4°C). The resulting pellets were resuspended in phosphate buffer (50 mM, pH 6.0) with 0.5% hexadecyltrimethylammonium bromide (Sigma). The suspension underwent four freeze-thaw cycles and then was briefly sonicated (10 sec). The samples were then centrifuged and the protein contents of the supernatants were determined (Pierce Micro BCA Protein Assay, Thermo Scientific, Waltham, MA). Equal amounts of protein from each sample were incubated with TMB (Enzo Life Science, Inc., Farmingdale, NY, USA) for 15 minutes at 37°C. The reaction was stopped with sulfuric acid and the absorbance was read at 405 nm.

### Statistical analyses

Individual studies included 3–15 mice per group, as reported in the figure legends. Results were compared using one-way or two-way ANOVA with Bonferroni post-hoc testing, or Mann-Whitney tests as appropriate for the data. *P* values < 0.05 were considered statistically significant.

## Results

### CD18-mutant mice have normal livers at baseline

**Fig 1:**
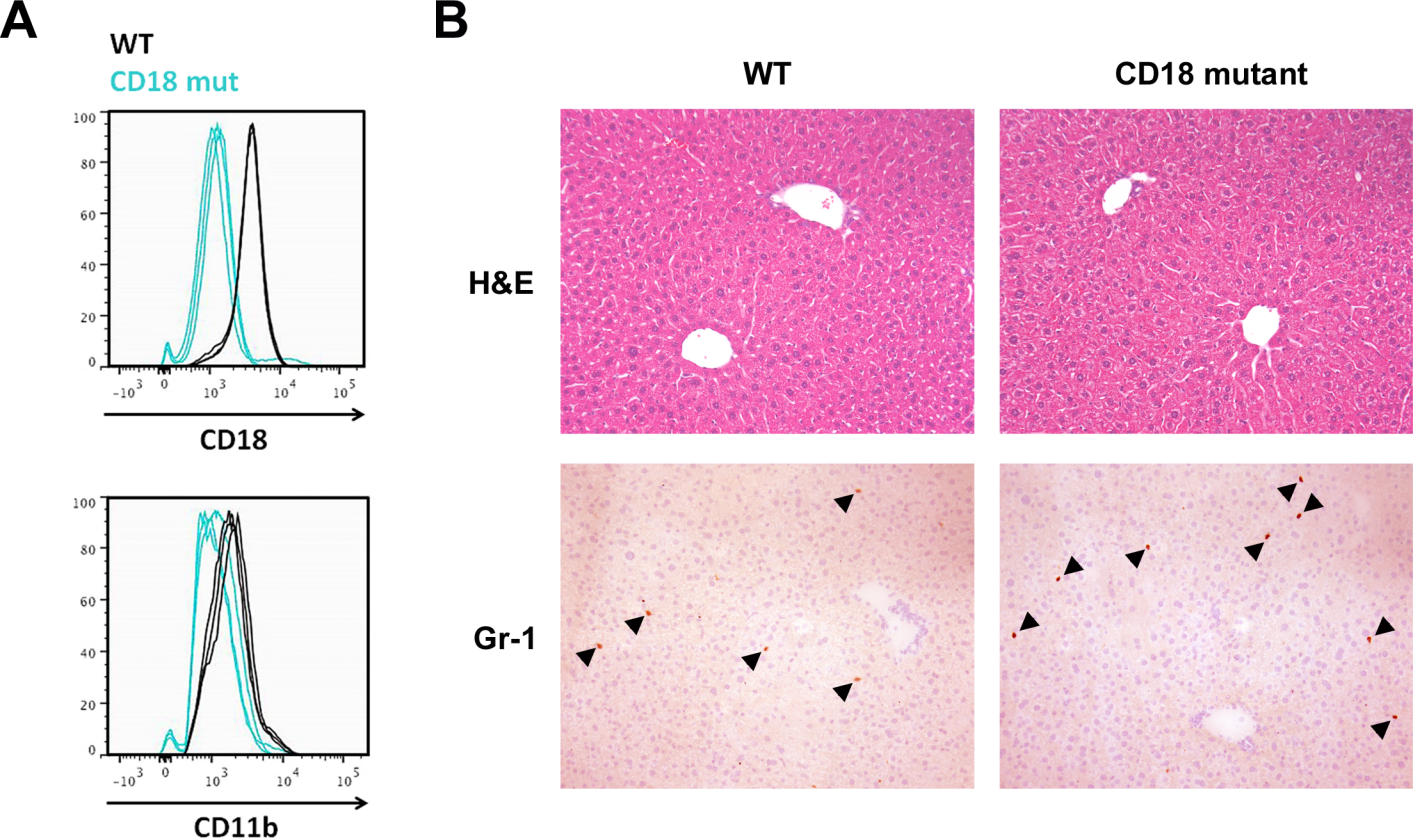
Comparative features of WT and CD18-mutant mice fed chow diets. (A) CD18 and CD11b expression measured by flow cytometry in leukocytes isolated from the livers of chow-fed WT and CD18-mutant (CD18 mut) mice. The fluorescence intensity of CD18 mut granulocytes was 70% lower than that measured in WT granulocytes, n = 3 per group. (B) Liver histology and Gr-1 immunohistochemistry in WT and CD18 mut mice. Arrowheads mark Gr-1-positive cells. For cell counts, see text. Original magnification 10X.

As expected, granulocytes isolated from the livers of CD18-mutant mice displayed weak CD18 staining (70% weaker than WT) as well as reduced immunoreactivity for the related β_2_-integrin subunit CD11b (Fig 1A). Under chow-fed conditions, CD18-mutant mice displayed normal liver histology, although mutant livers tended to contain more granulocytes than WT livers (5.4 ± 1.1 vs. 3.0 ± 0.5 cells per 10X field, *P* = 0.09, Fig 1B). Chow-fed CD18-mutant mice displayed no abnormality in serum ALT (65.3 ± 3.6 vs. 61.5 ± 6.5 IU/L, *P* > 0.05). Their hepatic triglyceride content was also indistinguishable from that of WT mice (5.6 ± 0.9 vs. 7.6 ± 0.8 mg/g liver, *P* > 0.05).

### MCD feeding induces severe hepatic steatosis but only moderate steatohepatitis in CD18-mutant mice

**Fig 2:**
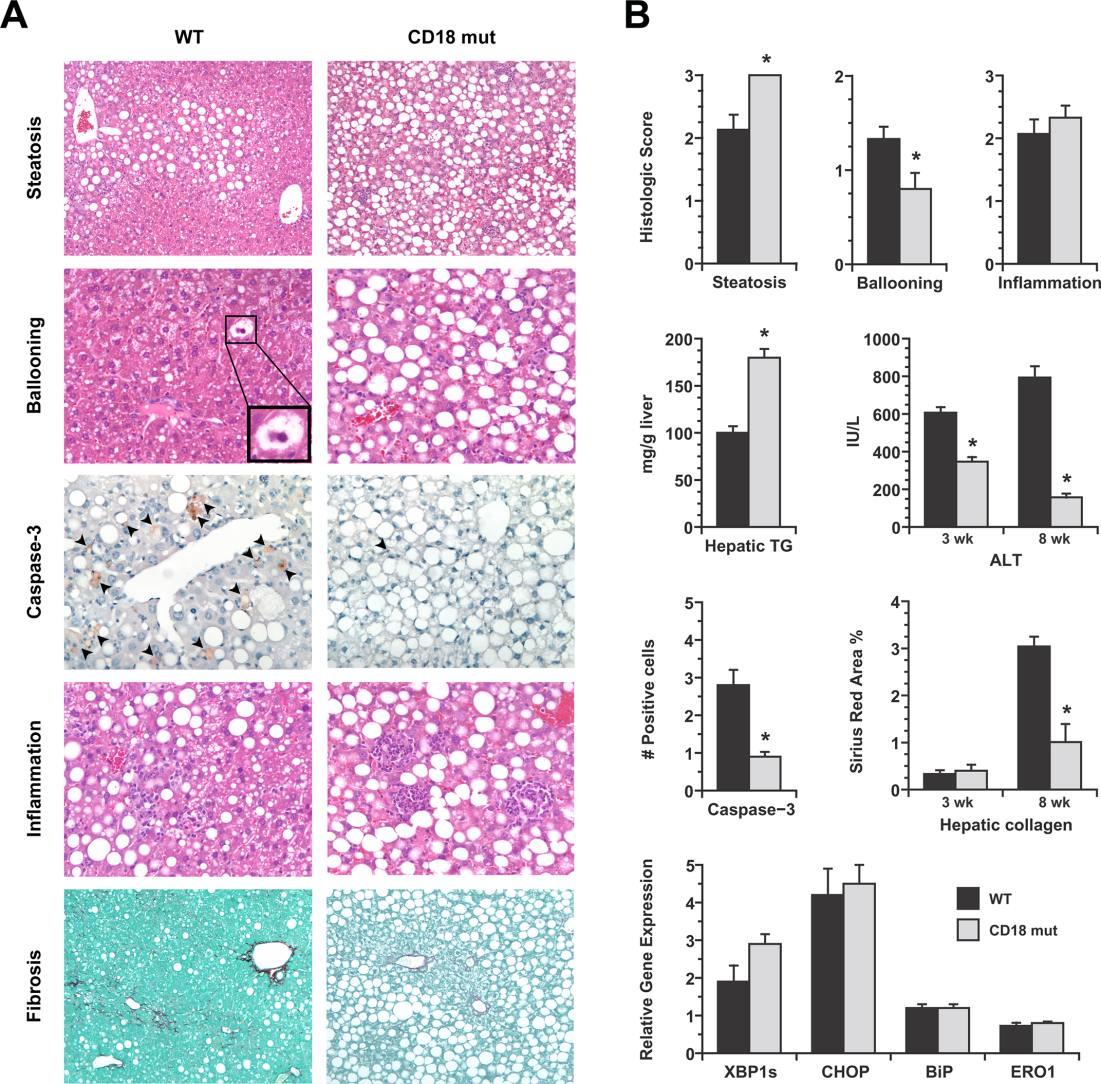
Comparative features of WT and CD18-mutant mice after MCD feeding. (A) Photomicrographs illustrate several features of liver disease in WT and CD18-mutant (CD18 mut) mice after MCD feeding for 3–8 wk. Steatosis (H&E, 3 wk, 10X) is more severe in CD18 mut livers whereas ballooning (H&E, 3 wk, 10X) is more evident in WT livers. WT livers also have more cells staining positively for cleaved caspase-3 (20X). Hepatic inflammation is visible in both WT and CD18 mut livers, although cells are diffusely distributed in WT livers and clustered in CD18 mut livers. Hepatic fibrosis (Sirius red, 8 wk, 10X) is more advanced in WT livers than CD18 mut livers. (B) Graphs illustrate comparative histologic scores for steatosis, ballooning and inflammation in WT and CD18 mut mice at 3 wk, hepatic triglyceride concentration at 3 wk, and serum ALT measurements at 3 wk and 8 wk. Additional histograms illustrate quantitation of caspase-3-positive cells (# cells per 20X field) and quantitation of hepatic fibrosis (Sirius red, percent area). Final histograms show hepatic mRNA levels for several ER stress markers in WT and CD18 mut liver, normalized to chow-fed WT liver. XBP1s, X-box protein-1 spliced form; CHOP, CEBP-homologous protein; BiP, binding immunoglobulin protein; ERO1, ER oxidoreductin. Values represent mean ± SE for n = 10–15. * *P* < 0.05 vs. WT.

WT and CD18-mutant mice both developed weight loss in response to MCD feeding, which is typical for this experimental model [14]. There was no difference between the two groups in food intake (2.9 ± 0.2 vs. 3.1 ± 0.2 g/d in WT vs. CD18 mutant, *P* > 0.05) or body weight change over the experimental feeding period (-28.8% ± 0.4% vs. -26.7 ± 1.7% at 3 wk, -42.3 ± 1.8% vs. -38.9 ± 1.1% at 8 wk in WT vs. CD18 mutant, *P* > 0.05). Both groups of mice also developed liver injury in response to MCD feeding; however, important differences in disease severity were noted between the two genotypes (Fig 2). The most obvious difference was that hepatic steatosis was more severe in CD18-mutant livers than WT livers (Fig 2A). Hepatic triglyceride content was nearly two times higher in the CD18-mutant group (179.9 ± 9.2 vs. 100.0 ± 7.0 mg/g liver, *P* = 1.4 x 10^−7^) at 3wk (Fig 2B). Despite enhanced steatosis, hepatocellular injury was milder in CD18 mutants than WT mice, demonstrated by less histologic ballooning of hepatocytes, fewer liver cells exhibiting cleaved caspase-3 and a 40% or greater reduction in serum ALT (Fig 2A and 2B). There was no difference in ER stress marker expression between WT and CD18-mutant mice to account for the difference in liver injury (Fig 2B). By 8 weeks of MCD feeding, WT mice displayed clear evidence of liver fibrosis whereas CD18-mutant mice did not (Fig 2A and 2B).

**Fig 3:**
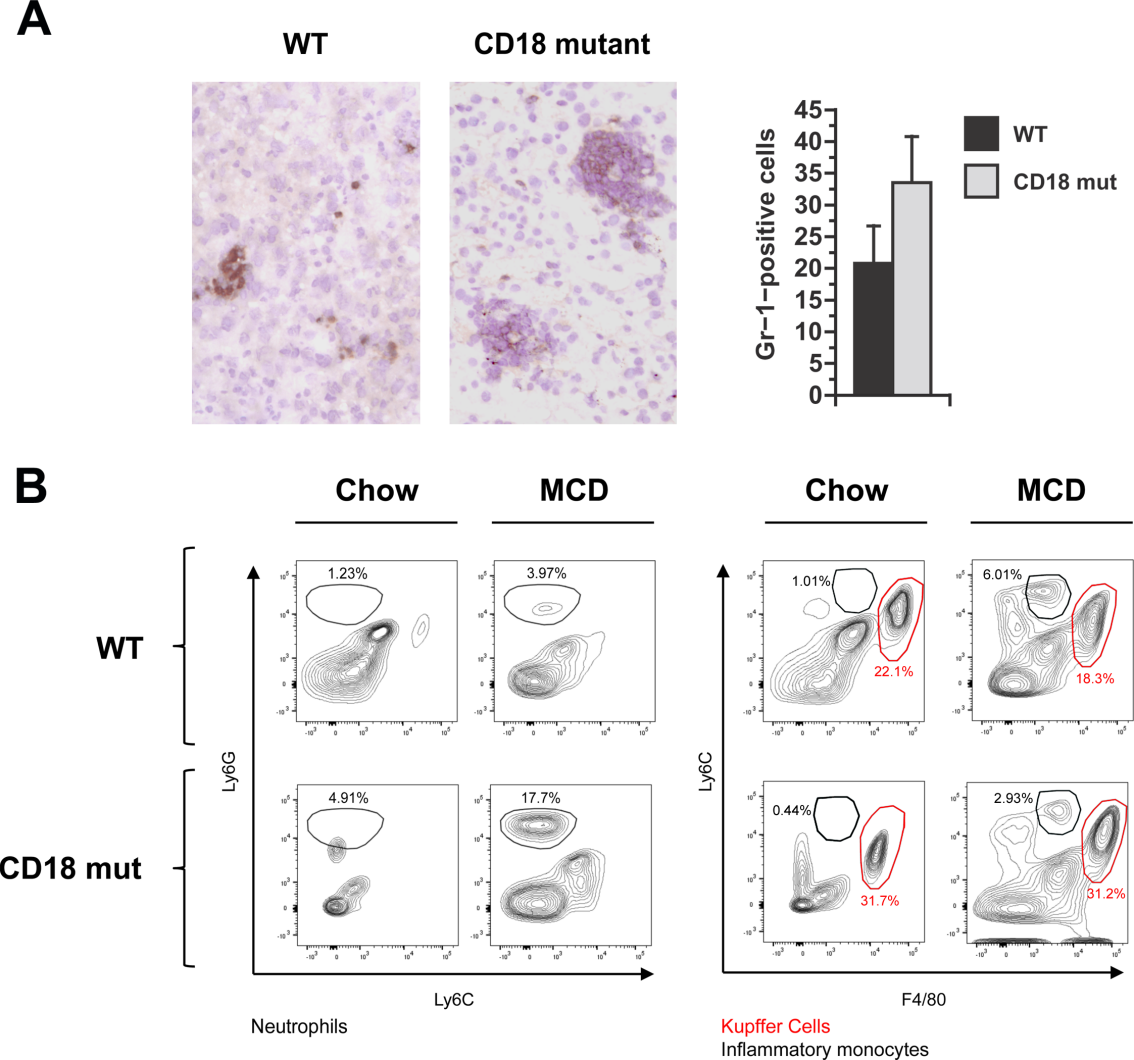
Hepatic inflammation in WT and CD18-mutant mice in response to MCD feeding. (A) Gr-1 staining and quantitation of Gr1-positive cells in WT and CD18 mut liver at 3 wk. Photomicrographs illustrate that Gr-1-positive cells are abundant in both WT and CD18-mutant (CD18 mut) mice after MCD feeding, although in different distributions. Original magnification 15X. Histograms illustrated Gr1-positive cell counts in WT and CD18 mut livers, performed as described in Methods. Values represent mean ± SE for n = 5. (B) Representative FACS plots of hepatic leukocytes from mice fed chow or MCD diets. MCD feeding enhances the proportion of neutrophils (Ly6G^high^) and inflammatory monocytes (Ly6C^high^), while the lack of CD18 further enhances the accumulation of neutrophils but not inflammatory monocytes. Data are illustrative of n = 6 mice per group (24 per cohort), performed as 2 replicate experiments involving 12 mice each (3 per group).

Hepatic leukocyte infiltration was evident in the livers of both WT and CD18-mutant mice. By histologic scoring, the degree of hepatic inflammation was comparable between the two groups (Fig 2B), but CD18 mutants displayed a unique pattern of inflammation characterized by prominent aggregates of inflammatory cells in the hepatic parenchyma (Figs 2A and 3A). Analysis of hepatic leukocytes by flow cytometry revealed that CD18-mutant mice accumulated more neutrophils in their livers than WT mice in response to MCD feeding (104,750 vs. 251,800 neutrophils in WT vs. CD18-mut, *P* = 0.04). Infiltration of inflammatory monocytes (Ly6C^hi^) was similar between the two groups (256,150 vs. 149,240 cells in WT vs. CD18-mut, *P* = 0.19) (Fig 3B). CD18 deficiency, therefore, did not impair the recruitment of either neutrophils or monocytes to the liver in response to MCD feeding.

**Fig 4:**
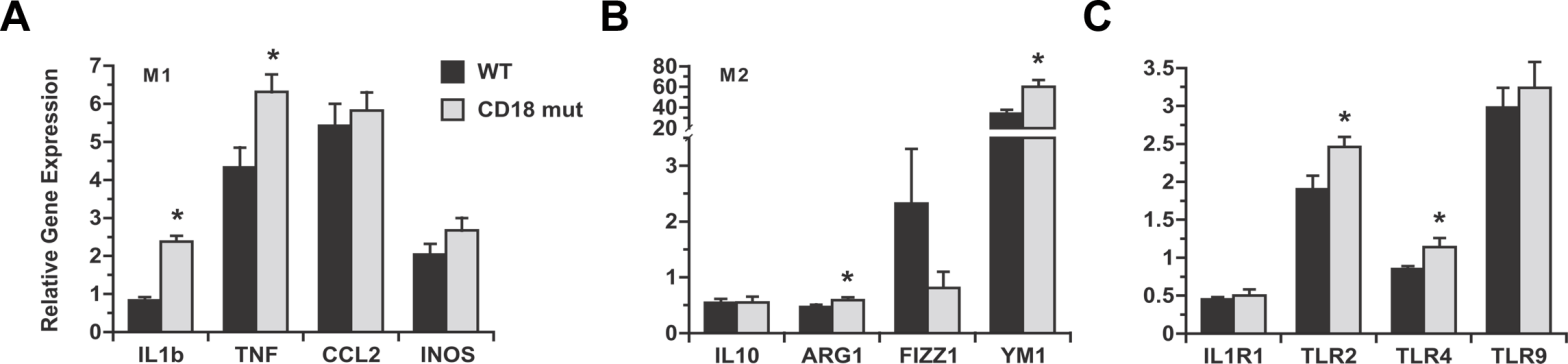
Comparative expression of cytokine and inflammatory genes in WT and CD18-mutant mice after MCD feeding. All data are normalized to the expression levels measured in WT mice on chow diets. (A) Hepatic expression of M1 inflammatory genes is up-regulated by MCD feeding in both WT and CD18 mut mice. M1 genes are induced as much or more in CD18-mutant mice than WT mice. M1 genes are induced as much or more in CD18-mutant mice than WT mice. CCL2, C-C chemokine ligand-2; INOS, inducible nitric oxidase synthase. (B) Hepatic expression of M2 inflammatory genes after MCD feeding for 3 wk. ARG1, arginase-1; FIZZ1, found in inflammatory zone protein; Ym1, chitinase 3-like-3. (C) Hepatic expression of IL-1 receptor-1 (IL1R1) is decreased, and Toll-like receptors (TLR) increased, by MCD feeding in WT and CD18-mutant mice. TLR induction is not impaired, and in some instances enhanced, in CD18-mutant mice. Values represent mean ± SE for n = 8–14. *P < 0.05 vs. WT.

**Fig 5:**
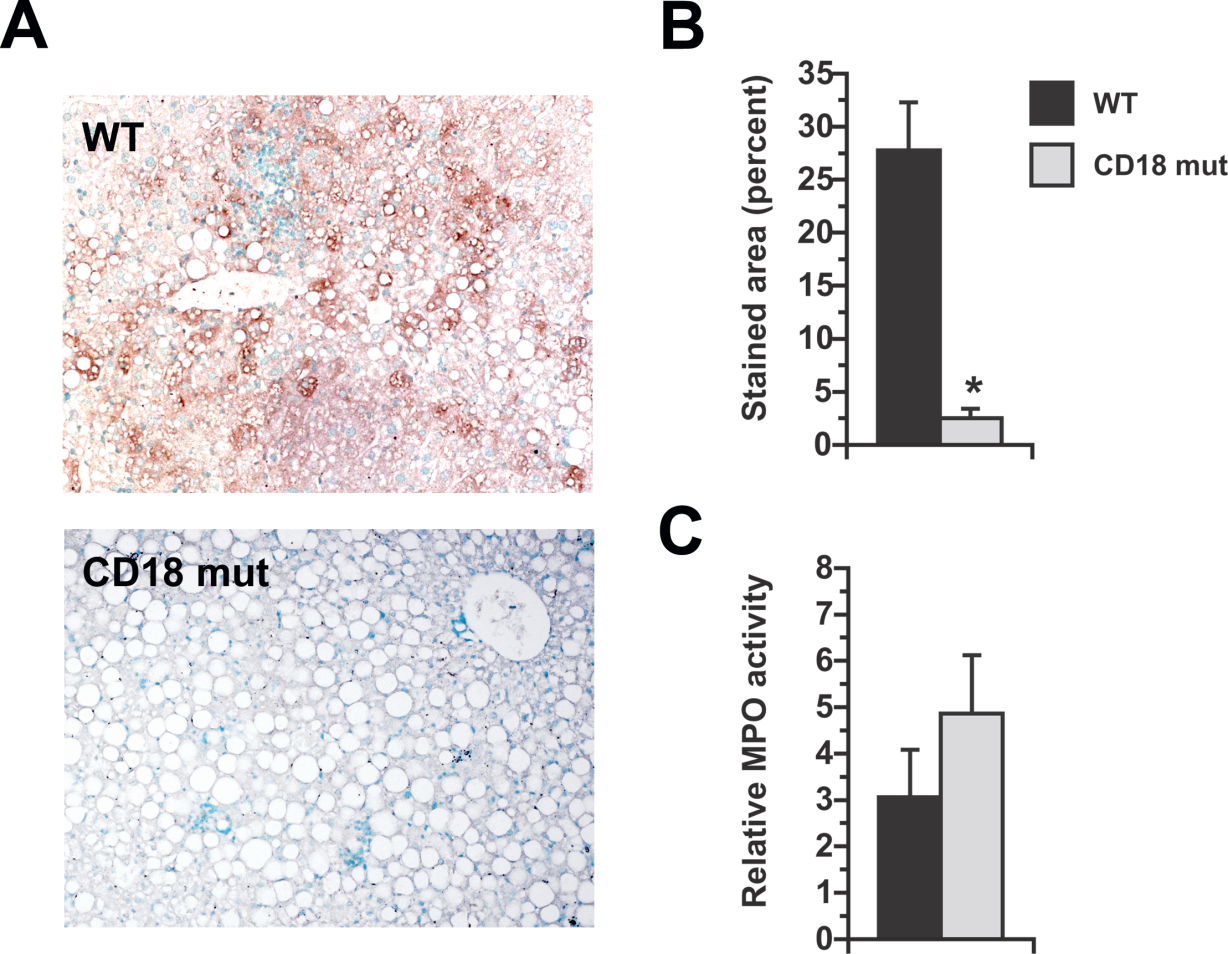
Chlorotyrosine-protein adduct formation in the livers of MCD-fed mice. (A) Immunohistochemical staining for chlorotyrosine-protein adducts in WT and CD18 mut mice fed MCD diets for 3 wk. Original magnification 15X. (B) Morphometric quantitation of adduct-stained area. (C) Liver myeloperoxidase (MPO) activity normalized to the level in chow-fed WT mice. Values represent mean ± SE for n = 5. *P < 0.05 vs. WT.

β_2_-integrins promote pro-inflammatory cytokine expression by leukocytes in addition to regulating cell migration. Notably, the influence of β_2_-integrins on cytokine induction can be independent of their effect on tissue invasion [26]. MCD feeding typically stimulates the expression of pro-inflammatory cytokine genes in the liver as it induces steatohepatitis [17, 27–30]; to determine whether CD18 influences inflammatory gene regulation in response to MCD feeding, we measured the levels of several pro- and anti-inflammatory genes in the livers of WT and CD18-mutant mice liver at 3 wk. We found that pro-inflammatory (M1) genes were similar or in some cases elevated in the CD18-mutant livers (Fig 4A). Anti-inflammatory (M2) genes were expressed at comparable levels in the livers of WT and CD18-mutant mice (Fig 4B). These results imply that CD18 is not required for MCD-mediated activation of pro-inflammatory signals in the liver and its absence does not alter the hepatic cytokine balance toward an anti-inflammatory phenotype. We also looked for alterations in hepatic expression of IL-1 receptor and TLRs in CD18-mutant mice after MCD feeding, as these have been implicated in the development of experimental steatohepatitis [5, 31–37]. Again, we found no reduction in these genes in CD18 mutants compared to WT mice (Fig 4C). Since CD18 deficiency failed to suppress any of these prerequisites to MCD-mediated steatohepatitis, but still reduced the severity of liver injury, we reasoned the improved liver outcome in CD18-mutant mice was due to impaired leukocyte function. Accordingly, we examined livers for chlorotyrosine-protein adducts, which are markers of leukocyte activation and oxidant release [38, 39]. Chlorotyrosine-protein adducts were abundant in WT livers but not in CD18-mutant livers, even in the vicinity of large leukocyte aggregates (Fig 5A and 5B). This was true despite comparable hepatic levels of myeloperoxidase, signifying that inflammatory cells of both genotypes are equipped with the machinery necessary to produce superoxide and hypochlorous acid [40, 41] (Fig 5C). Overall, the data indicate that in the MCD model of steatohepatitis, CD18 deficiency does not interfere with events that attract leukocytes to the liver, but does prevent recruited leukocytes from contributing to steatohepatitis. Indeed, they demonstrate that a substantial proportion of the liver injury that occurs in response to MCD feeding is a consequence of leukocyte-mediated damage to hepatocytes.

### Hepatic lipid metabolism in WT and CD18-mutant mice

**Fig 6:**
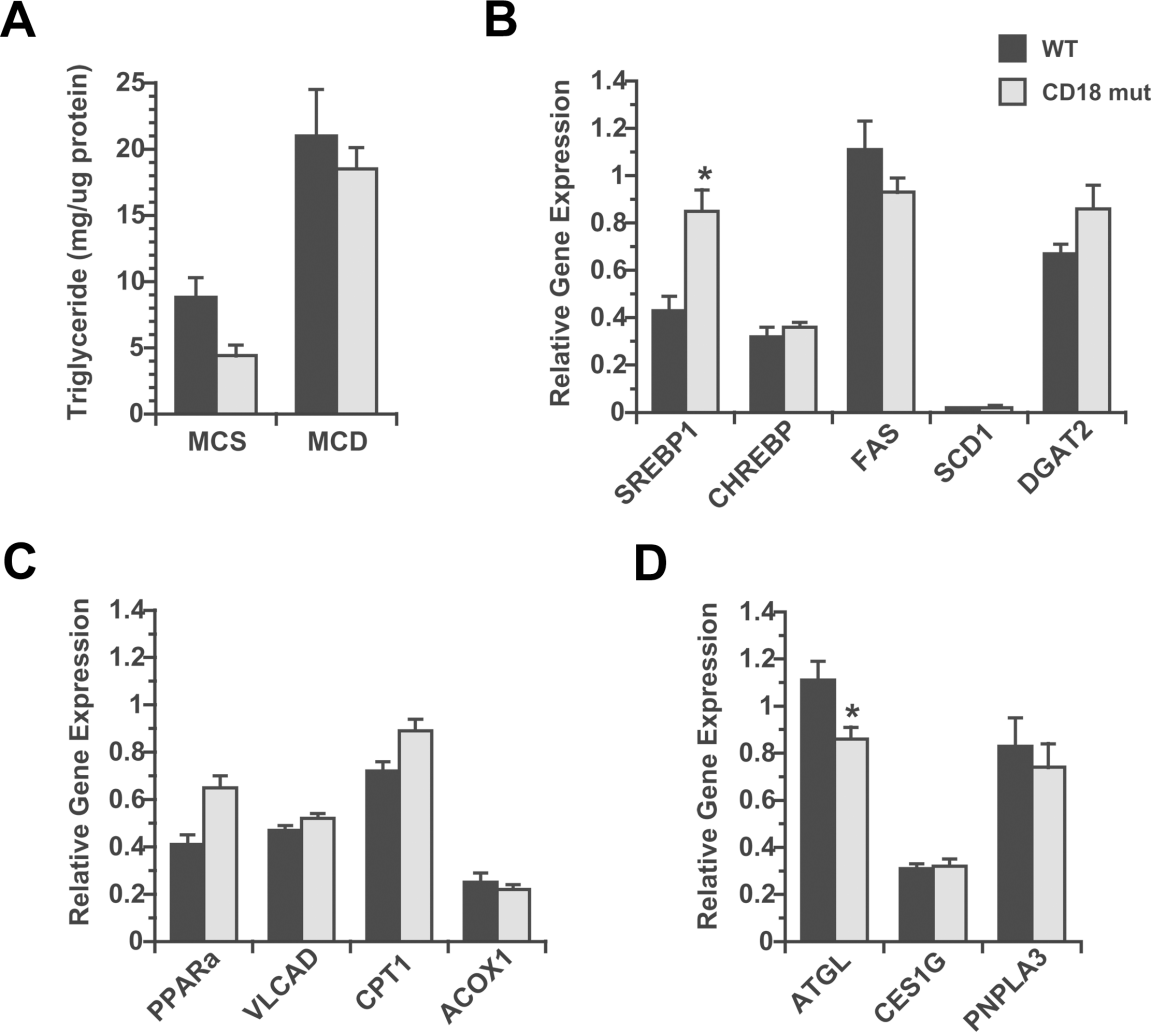
Analysis of hepatic lipid homeostasis in response to MCD in culture and in vivo. (A) Hepatic lipid accumulation in primary hepatocytes from chow-fed WT and CD18 mut mice, cultured in methionine-choline-sufficient (MCS) or methionine-choline-deficient (MCD) medium for 48 h. Values represent mean ± SE for n = 3. (B, C, D) Hepatic expression of genes pertinent to lipogenesis (B), fatty acid oxidation (C) and lipolysis (D) in WT and CD18 mut mice after 3 wk of MCD feeding in vivo, normalized to WT mice on chow diets. ACOX1, acyl-CoA oxidase-1; ATGL, adipose triglyceride lipase; CPT1, carnitine palmitoyl transferase-1; DGAT2, diacylglycerol transferase-2; FAS, fatty acid synthase; PNPLA3, patatin-like phospholipase domain containing 3; PPARa, peroxisome proliferator activated receptor-α; SCD1, stearoyl-CoA desaturase-1; VLCAD, very long chain acyl dehydrogenase. Values represent mean ± SE for n = 8–14. * *P* < 0.05 vs. WT.

In an effort to explain why CD18-mutant mice developed exaggerated hepatic steatosis in response to MCD feeding, we isolated hepatocytes from chow-fed WT and CD18-mutant mice and cultured them for 48 h in MCD medium to induce lipid accumulation [42]. Mutant hepatocytes developed no more steatosis than WT hepatocytes, indicating that the tendency toward enhanced steatosis in vivo is not hepatocyte-intrinsic (Fig 6A). After MCD feeding in vivo, hepatic expression of IL-1 and TNF were significantly increased in CD18-mutant livers (Fig 4A), which could stimulate hepatic lipogenesis. Indeed, we found that mRNA encoding the lipogenic transcription factor SREBP1 was twice as abundant in CD18-mutant as WT mice (Fig 6B). By contrast, genes involved in fatty acid oxidation were largely similar in WT and CD18-mutant livers (Fig 6C). Investigating lipid hydrolysis, we found that mRNA encoding adipose triglyceride lipase (ATGL) was moderately but significantly reduced in the livers of CD18-mutant mice relative to WT mice after MCD feeding (Fig 6D). This suggests that slowed triglyceride lipolysis may also contribute to enhanced hepatic steatosis in the setting of CD18 deficiency.

## Discussion

CD18 is a key mediator of neutrophil and macrophage activation in tissue injury. In this study, we used CD18-mutant mice to elucidate the contribution of activated neutrophils and macrophages to experimental steatohepatitis induced by an MCD diet. Our results confirmed our initial hypothesis that CD18-mutant mice would be protected from MCD-mediated liver injury; importantly, though, the protection was restricted to certain features of steatohepatitis. Hepatocyte injury was clearly reduced in CD18-mutant mice. By contrast, hepatic inflammation was not, and hepatic steatosis was actually worse than in WT mice. These findings indicate that CD18 is not necessary for the development of several MCD-related abnormalities including steatosis, pro-inflammatory cytokine induction and leukocyte recruitment. Rather, CD18 plays a singular role in leukocyte activation in MCD-fed mice, which by itself contributes significantly to their overall liver outcome.

CD18 is the molecule that enables leukocytes to adhere firmly to vascular endothelial cells before migrating out of the circulation into sites of tissue injury [43]. Accordingly, one might expect CD18-mutant mice to exhibit less hepatic inflammation in response to MCD feeding. This was not the case in our experiments: indeed, CD18-mutant mice had hepatic inflammation scores and leukocyte counts comparable to those in WT mice after 3 wk of MCD exposure. The limited impact of the CD18 mutation on hepatic inflammation may be attributable to low-level expression of CD18 on leukocytes (Fig 1), which could be sufficient to support their transmigration into the liver. Alternatively, infiltration of leukocytes into MCD-fed livers could be CD18-independent, which is known to occur [10, 20, 44–47]. Whatever the reason, despite the fact that hepatic inflammation was not impaired in CD18-mutant mice, it was accompanied by far less liver injury than in WT mice. This is consistent with the notion that activation of innate immune cells, which is impaired in CD18 mutants, is a key event in MCD-mediated steatohepatitis. Leukocyte-mediated death of hepatocytes is an important event in other liver diseases such as hepatic ischemia-reperfusion injury [48, 49] and certain drug-induced and cholestatic disorders [20, 50, 51]. In these situations, engagement of the CD11/CD18 receptor on leukocytes helps trigger a respiratory burst that results in the formation of superoxide and hypochlorous acid, which damages adjacent cells [52–54]. Leukocyte-derived proteases can also mediate hepatotoxicity by killing hepatocytes directly or activating cytokines and growth factors to exacerbate inflammation [54–56]. In the current study, it was the inability of CD18-mutant leukocytes to generate toxic oxidants in the liver that coincided directly with protection from steatohepatitis. This underscores the importance of activated innate immune cells to the pathogenesis of fatty liver disease.

Our experiments place CD18 alongside other immunoreactive molecules that influence the development of steatohepatitis. Just as CD18-mutant mice are protected against MCD-mediated liver injury, mice with targeted disruption of genes encoding pro-inflammatory cytokines, cytokine receptors, and TLRs are also able to resist experimental fatty liver disease [28, 31, 57–59]. Many of these mice exhibit global reductions in all features of steatohepatitis, including steatosis, inflammation and hepatocellular injury, which is distinct from the outcome we observed in CD18-mutant mice. In these other strains, Kupffer cells have been implicated as key mediators of adverse liver outcomes [5, 29, 37, 60]. Interestingly, CD18-mutant mice showed no evidence of Kupffer cell dysfunction after MCD feeding. This important difference may account for the more specific yet still significant impact of CD18 deficiency on liver-related outcome. Mice deficient in CC-chemokine receptor-2 (CCR2), which prevents leukocyte recruitment to the liver, should theoretically exhibit a similar pattern of protection against steatohepatitis as CD18-mutant mice, because both have defects affecting events in the liver downstream of Kupffer cell activation. This is unfortunately difficult to confirm, because the protection afforded by CCR2 deficiency against steatohepatitis varies depending on the model used to induce liver injury [30, 59, 61]. Even so, our observations by themselves underscore the specific role played by newly infiltrated leukocytes in the pathogenesis of steatohepatitis. They indicate that as much as 40% of the liver injury in the MCD model is attributable to recruited leukocytes, whereas the remaining features of steatohepatitis arise from liver-autonomous events.

One intriguing finding in our study was that MCD feeding exaggerated hepatic steatosis in CD18-mutant mice. Since hepatocytes from WT and CD18-mutant mice responded identically to methionine and choline deprivation in culture (Fig 6A), leukocytes are likely driving the steatotic phenotype in CD18-mutant mice in vivo. There is a precedent to this concept, as mice lacking leukocyte adhesion molecules or certain NADPH oxidases have a reported propensity toward diet-induced obesity and alterations in hepatic lipid metabolism [62–64]. Even humans with a polymorphism in ITGB2 that results in reduced CD18 expression are at increased risk of obesity [65]. Taken together, these observations imply that activated leukocytes impose some tonic influence on metabolism that actually limits fat accumulation in hepatocytes. Applying this concept to human NASH, it is possible that prolonged hepatic inflammation is responsible for the decline in hepatic steatosis that occurs during disease progression [66–69].

Our work did not pinpoint a single dominant mechanism by which CD18 deficiency influenced hepatic lipid metabolism. CD18 mutants did exhibit increased IL-1 and TNF gene expression in the liver, which has been linked to hepatic steatosis [33, 60, 70]. We also found reduced ATGL gene expression in CD18-mutant mice. Global ablation of ATGL in mice exacerbates MCD-mediated hepatic steatosis [71], and liver-specific ablation of this enzyme leads to steatosis even on a normal diet [72]. It is possible that enhanced hepatic cytokine expression in CD18-mutant mice, combined with suppressed hepatic lipolysis, results in enhanced hepatic steatosis.

In conclusion, the current experiments demonstrate that CD18-mediated leukocyte activation is a central event in the pathogenesis of steatohepatitis in response to an MCD diet. Specifically, activated leukocytes recruited to the liver in response to MCD feeding greatly amplify the degree of hepatocellular injury in this NASH model. Events upstream of leukocyte activation remain largely unperturbed in CD18-mutant mice; this permits a distinction between liver outcomes that are either dependent on or independent of innate immune cell invasion. Interestingly, our results suggest that recruited leukocytes not only promote liver cell injury, but also modulate hepatic steatosis. This may be mediated by dual effects on hepatic lipogenesis and lipolysis.

## Supporting information

S1 Table NC3Rs ARRIVE guidelines checklist. (PDF)

## Abbreviations

ACOX1: acyl-CoA oxidase-1
ARG1: arginase-1
ATGL: adipose triglyceride lipase
ALT: alanine aminotransferase
BiP: binding immunoglobulin protein
CCL2: C-C chemokine ligand-2
CCR2: CC-chemokine receptor-2
CES1G: carboxylesterase 1G
CD18: mut, CD18-mutant
CHOP: CEBP-homologous protein
CHREBP: carbohydrate responsive element-binding protein
Col1a1: type I collagen α-1 chain
CPT1: carnitine palmitoyl transferase-1
DGAT2: diacylglycerol transferase-2
ERO1: ER oxidoreductin
FAS: fatty acid synthase
FATP5: fatty acid transport protein 5
FIZZ1: found in inflammatory zone protein
IL1R1: IL-1 receptor-1
INOS: inducible nitric oxide synthase
MCD: methionine-choline-deficient
MCS: methionine-choline-sufficient
MPO: myeloperoxidase
NASH: non-alcoholic steatohepatitis
PNPLA3: patatin-like phospholipase domain containing 3
PPARa: peroxisome proliferator activated receptor-α
qPCR: quantitative PCR
SCD1: stearoyl-CoA desaturase-1
SREBP1: sterol regulatory element binding-protein 1
TLR: Toll-like receptor
VLCAD: very long chain acyl dehydrogenase
WT: wild-type
XBP1s: X-box protein-1 spliced form
YM1: chitinase 3-like-3
